# Therapeutic Horizon in Multiple Myeloma: Analysis of the Emerging Landscape of Clinical Trials

**DOI:** 10.1007/s43441-026-00971-7

**Published:** 2026-05-07

**Authors:** Marina Alacoque Rodrigues, Cristiane Aparecida Menezes de Pádua, Paula Lana de Miranda Drummond, Jéssica Soares Malta, Adriano Max Moreira Reis

**Affiliations:** 1 College of Pharmacy, Universidade Federal de Minas Gerais, 6627 Antonio Carlos Ave, Belo Horizonte, Minas Gerais 31270010 Brazil; 2 Fundação Ezequiel Dias, Belo Horizonte, Minas Gerais Brazil

**Keywords:** Multiple myeloma, Clinical trials, Therapeutics, Immunotherapies

## Abstract

**Background:**

Emerging therapies for multiple myeloma (MM) have significantly improved patient outcomes extending survival and advancing treatment toward more targeted, effective, and less toxic approaches. However, the disease remains incurable.

**Objectives:**

Analyze the therapeutic landscape of MM by evaluating drug candidates in clinical trials from 2014 to 2024.

**Methods:**

Data were extracted from ClinicalTrials.gov (CTG) and Cortellis Drug Discovery Intelligence (CDDI), including active and completed studies with results on newly diagnosed and refractory MM. Joinpoint regression models estimated Annual Percent Changes (APCs) and Average Annual Percent Changes (AAPC). Data were examined by clinical trial characteristics, targeting strategies, and potential impact on treatment advancements.

**Results:**

A total of 1091 trials from CDDI and 1947 from CTG were screened, yielding 365 studies eligible for detailed analysis. Phase I trials were the most common (*n* = 182; 49.9%), and biologic agents represented the majority of the investigational therapies (*n* = 251; 68.8%). Among intervention types, cell therapies were predominant (*n* = 171; 46.8%), with CAR-T products targeting BCMA (*n* = 107), CD19 (*n* = 16), and GPRC5D (*n* = 12) emerging as the most studied in recent years. Before the joinpoint, the number of active trials grew by 1.86% per year (95% CI: 1.67–2.06), corresponding to an average increase of 11.9 studies annually. After the joinpoint, growth accelerated to 3.69% per year (95% CI: 3.19–4.19), equivalent to 26.5 additional trials each year. Across the entire study period, the weighted average annual percentage change (AAPC) was 2.63%.

**Conclusion:**

The rise in MM clinical trials highlights a shift toward biologic therapies, particularly immunotherapies and gene therapies like CAR-T.

## Introduction

Multiple myeloma (MM) is a hematological malignancy originating from the uncontrolled proliferation of abnormal plasma cells in the bone marrow. It is the second most prevalent type of blood cancer, with an increasing global incidence, particularly among men, individuals aged 50 and older, and populations in high-income countries [1–3].

Despite substantial advancements in treatment, MM remains incurable, necessitating continued innovation in therapeutic strategies [4]. The disease imposes a significant symptom burden and is associated with the lowest health-related quality of life (HRQoL) among hematological malignancies [5]. Recent approvals by the U.S. Food and Drug Administration (FDA) of idecabtagene vicleucel (ide-cel) and ciltacabtagene autoleucel (cilta-cel) have transformed the treatment landscape, paving the way for cellular therapies that harness natural killer (NK) and dendritic cells (DCs) [6]. Currently approved treatments also include immunomodulatory drugs (IMiDs), proteasome inhibitors (PIs), monoclonal antibodies (mAbs), and bispecific antibodies (BsMAb) [7].

Emerging therapies increasingly target key molecular markers such as B-cell maturation antigen (BCMA), G protein-coupled receptor class C group 5 member D (GPRC5D), Fc receptor-homolog 5 (FcRH5), cluster of differentiation 38 (CD38), signaling lymphocytic activation molecule F7 (SLAMF7), and B-cell lymphoma 2 (BCL-2) [8]. However, patients with high-risk MM, those with adverse cytogenetics, Stage III disease according to the Revised International Staging System (R-ISS), or resistance to standard therapies—continue to face poor prognoses, representing a critical unmet need [9].

Previous studies have focused on promising therapies for MM by analyzing specific therapeutic targets and the distinct characteristics of individual agents [8–10]. In contrast, this study aimed to provide an overview of the evolution of innovative, disease-modifying therapies in MM by mapping trends in trial registrations, therapeutic strategies, investigational drug classes, and trial design features. By tracking the evolution of research in this field, our analysis seeks to inform therapeutic decision-making and guide the development of innovative treatment strategies to improve patient outcomes.

## Methods

### Data Sources and Processing

This study employed data extraction from two online databases, ClinicalTrials.gov (CTG), available at https://clinicaltrials.gov/, and Cortellis Drug Discovery Intelligence (CDDI), available at https://www.cortellis.com/drugdiscovery. CTG is a publicly accessible, internet-based registry managed by the U.S. National Library of Medicine. As one of the most comprehensive clinical research databases globally, it is widely used to characterize study populations and identify trends in clinical research and treatment approaches [11–13]. The CDDI, maintained by Clarivate Analytics, is a global intelligence database covering clinical trials for drugs, biologics, diagnostics, and biomarkers [14].

The search period covered all studies registered between January 1, 2014, and December 31, 2024. For the search strategy in CTG, the keyword “Multiple Myeloma” was used in the “Condition or Disease” section. In CDDI, the search was conducted using the “Advanced Search” tool, with the filters: “Clinical Studies,” “Condition”―using the keyword Multiple Myeloma―and “Available Since.” No recruitment status limit was applied for either search, ensuring that all available clinical trials were selected. Data were collected in February 2025.

Our analysis intentionally focuses on innovative, disease-modifying therapies in MM. Therefore, trials involving drugs without direct anti-tumor activity (e.g., anesthetics or supportive care agents), as well as reformulations, biosimilars, label extensions, and diagnostic or prevention studies, were excluded. Trials investigating novel agents in combination with an approved backbone regimen were retained to capture first-in-class and truly innovative compounds. Importantly, for therapies that eventually became approved, all their clinical trials initiated within our study period (2014–2024) were included and recorded according to their start date and the years they remained active prior to regulatory approval.

Data from CTG and CDDI were first processed separately to retain only unique NCT (National Clinical Trial number) within each database, ensuring there were no duplicates within a single registry. To ensure data accuracy and usability, selected data were manually reviewed and refined in multiple steps by two independent reviewers.

To define the dataset of unique trials, with active ongoing clinical trials evaluating new therapeutic strategies in a horizon-scanning context, data from CTG and CDDI were combined into a single dataset. Records sharing the same NCT number across the databases were consolidated into a single entry to prevent double counting between sources. The resulting dataset was subsequently filtered to include only active, ongoing studies investigating novel agents without prior market authorization, which constituted the final set of unique trials. The collected data was tabulated using Microsoft Excel 365 for Windows (Microsoft Press, Redmond, WA, USA).

### Statistical Analysis

Statistical analyses were performed at the trial level, with each trial considered an observation. Descriptive analyses included absolute and relative frequencies, with trial factors summarized by frequency and percentage. The number of clinical trials registered each year was analyzed to gain insights into registration trends over the study period. These data were plotted along a time axis to visualize the registration trend over the past decade.

To evaluate temporal trends in clinical trial registrations for MM, Joinpoint regression was employed. This method fits a segmented (piecewise) linear regression model, allowing the identification of time points (joinpoints) at which significant changes in trends occur in MM trial registrations over time [15]. Each segment is characterized by its own Annual Percent Change (APC), while the Average Annual Percent Change (AAPC) summarizes the overall trend across the entire study period [16]. The APC from year t to year (t + 1) is calculated using the following formula:

$$\:APC\left(\%\right)=\frac{{R}_{\left(t+1\right)}-\:{R}_{t}}{{R}_{t}}\:\times\:100\:=\:\left({e}^{\alpha}-1\right)\times\:100$$

Where R_t_ is the rate in year t and *α* is the slope coefficient in the linear equation below:

$$\:\mathrm{ln}({R}_{t})=\:\alpha\:t\:+\:\beta\:$$

The trend was considered increasing when the APC showed a positive result and the lower bound of the 95% confidence interval (CI) was greater than zero and decreasing when the APC showed a negative result and the upper bound of the 95% CI was below zero. Stability was defined when the 95% CI included zero. The AAPC was computed as a weighted average of the APC’s from the joinpoint model. For the trend analysis, studies were counted in the year(s) in which they were actively ongoing, in the CTG registry, for each calendar year. This approach was adopted because complete longitudinal trial status information was not consistently available in the CDDI database. As an additional analysis, negative binomial regression models were fitted to evaluate the association between trial phase and calendar year with the number of newly initiated and active trials reported in the CTG registry, accounting for overdispersion in count data. All statistical analyses were conducted using Python (version 3.12.6) with the SciPy library (version 1.15.1).

## Results

### Landscape of Emerging Clinical Trials in Multiple Myeloma

**Fig. 1:**
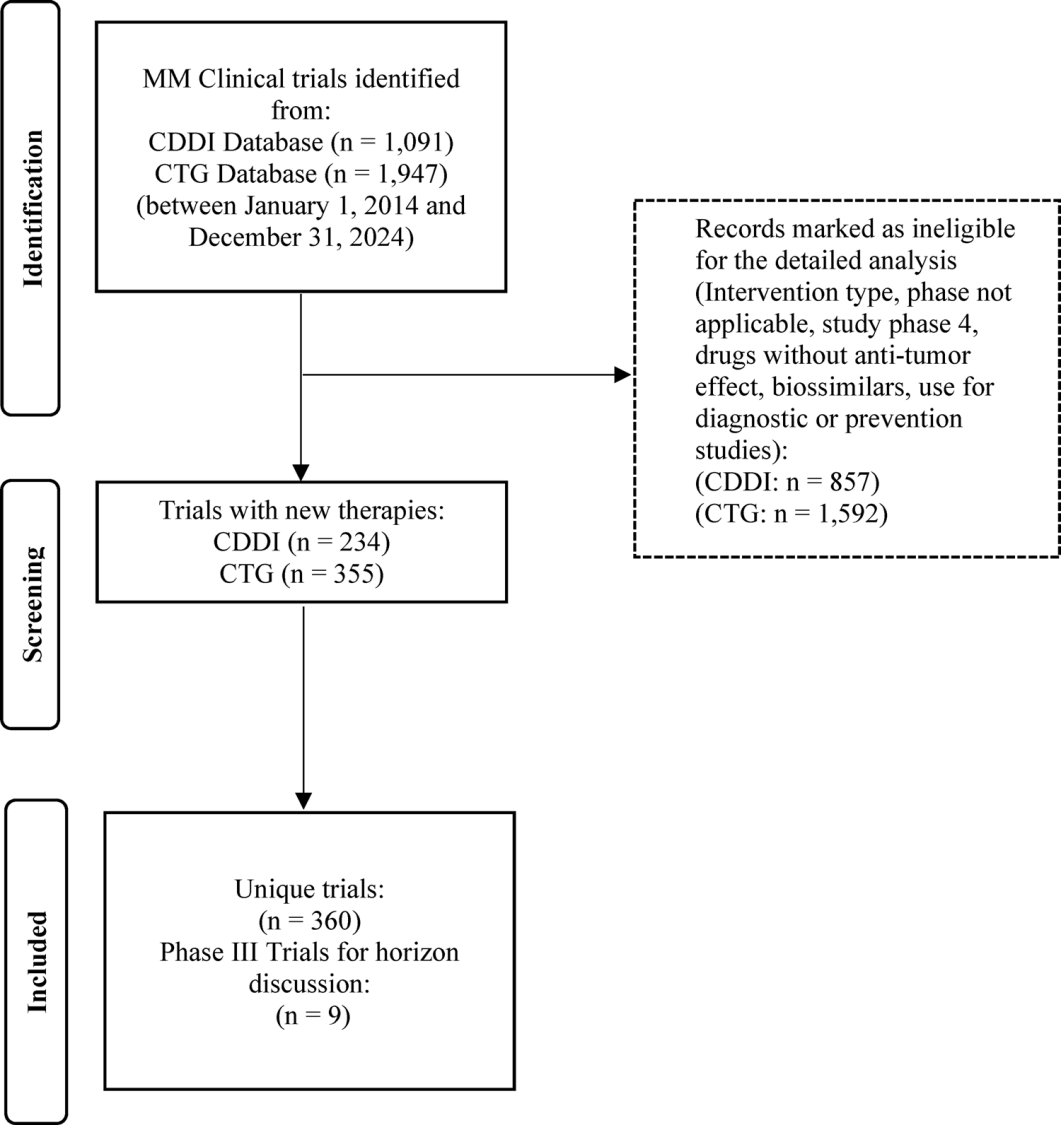
Flowchart of study selection for the analysis of MM clinical trials (2014–2024) from ClinicalTrials.gov Registry (CTG) and Cortellis Clinical Trials Intelligence (CDDI) databases

From January 1, 2014, to December 31, 2024, a total of 1091 clinical trials were retrieved from the CDDI database, and 1947 trials from the CTG database, following the predefined eligibility criteria, restricted to all active studies classified as early phase I, phase I, phase I/II, phase II, phase II/III, or phase III and with therapeutic intent. The detailed study selection process is illustrated in Fig. 1.

The analysis of both datasets revealed a marked predominance of open-label designs, encompassing over 90% of the identified MM studies, 96.4% in the CDDI and 97.3% in the CTG database.

In terms of study design characteristics within the CTG database, single-group assignment was observed in 19.5% (*n* = 378) of trials, whereas parallel assignment designs comprised 13.4% (*n* = 260). Unspecified assignments design comprised 67.3% (*n* = 1,309). Similar results were identified in the CDDI database. Phase I studies represented the majority of records across both databases, followed by Phase II and Phase III trials. Additional details regarding trial design modalities are provided in Table 1.

**Table 1:** Characteristics of MM clinical trials in CTG and CDDI databases (2014-24)

Clinical trial characteristics	Number (%)	
	CTG (*N* = 1947)	CDDI (*N* = 1091)
*Study phase*		
Early Phase	75 (3.9)	19 (1.7)
Phase I	535 (27.5)	307 (28.1)
Phase I/ II	336 (17.3)	161 (14.8)
Phase II	744 (38.2)	459 (42.7)
Phase II/III	16 (0.8)	10 (0.9)
Phase III	205 (10.5)	128 (11.7)
*Randomization*		
RCT	260 (19.5)	281 (25.8)
Non- RCT	378 (13.4)	
Unspecified	1309 (67.3)	810 (74.2)
*Blinding*		
Open label	1895 (97.3)	1052 (96.4)
Blinded	52 (2.7)	39 (3.6)

CTG, ClinicalTrials.gov Registry; CDDI, Cortellis clinical trials intelligence; RCT, Randomized controlled trial; MM, Multiple myeloma

Regarding regulatory sponsorship, approximately 39% of trials were registered with industry as the sponsor. Academic institutions accounted for 52.3% of trials in the CDDI and 53.9% in the CTG database. Contributions from the National Institutes of Health (NIH) or analogous governmental bodies represented 4.9% and 4.1% of studies in the CDDI and CTG databases, respectively. Other sponsor sources accounted for 3.8% of identified MM trials in CDDI and 3.1% in CTG.

Of the 221 studies identified as Phase III or Phase II/III in the CTG, considered in more advanced stages, 53% (*n* = 117) described eligible populations broadly classified as MM, without clear specification of treatment line, 20.4% (*n* = 45) reported newly diagnosed disease, and 10% (*n* = 22) explicitly defined relapsed or refractory multiple myeloma (RRMM) in their eligibility criteria. Among the trials identified in the CDDI, 64.5% (*n* = 89) did not specify treatment line, 13% (*n* = 18) focused on newly diagnosed disease, and 22.5% (*n* = 31) focused on RRMM.

The number of newly initiated MM clinical trials per year from 2014 to 2024, retrieved from both databases, is summarized in Table 2. In the CTG, annual trial counts ranged from 140 in 2021 to 108 in 2024, while in the CDDI they ranged from 115 in 2014 to 80 in 2023, with both datasets showing some fluctuation over the decade.

**Table 2:** Annual distribution of newly initiated clinical trials for MM by phase (2014–2024)

Year	CDDI					CTG				
	Study phase					Study phase				
	Phase I*	Phase I/II	Phase II**	Phase III	Total (*N*)	Phase I*	Phase I/II	Phase II**	Phase III	Total (*N*)
2014	29	15	57	14	115	44	15	47	13	119
2015	27	13	37	11	88	34	19	48	11	112
2016	28	14	45	6	93	32	17	53	10	112
2017	33	17	43	13	106	41	24	52	13	130
2018	35	13	47	10	105	46	22	53	14	135
2019	26	16	38	14	94	46	16	46	13	121
2020	29	11	53	10	103	59	13	54	12	138
2021	32	20	42	9	103	49	27	55	9	140
2022	38	19	33	19	109	57	21	37	18	133
2023	21	11	40	8	80	36	29	48	10	123
2024	28	12	41	14	95	41	18	35	14	108

Number (N) of newly initiated studies in the databases, stratified by study phase and by year. MM, Multiple Myeloma; CDDI, Cortellis Clinical Trials Intelligence; CTG, ClinicalTrials.gov Registry

* Studies classified as Early Phase I or Phase I

**Studies classified as Phase II/III represented fewer than 10 studies annually in our sample and were aggregated with Phase II for analytical purposes

**Fig. 2:**
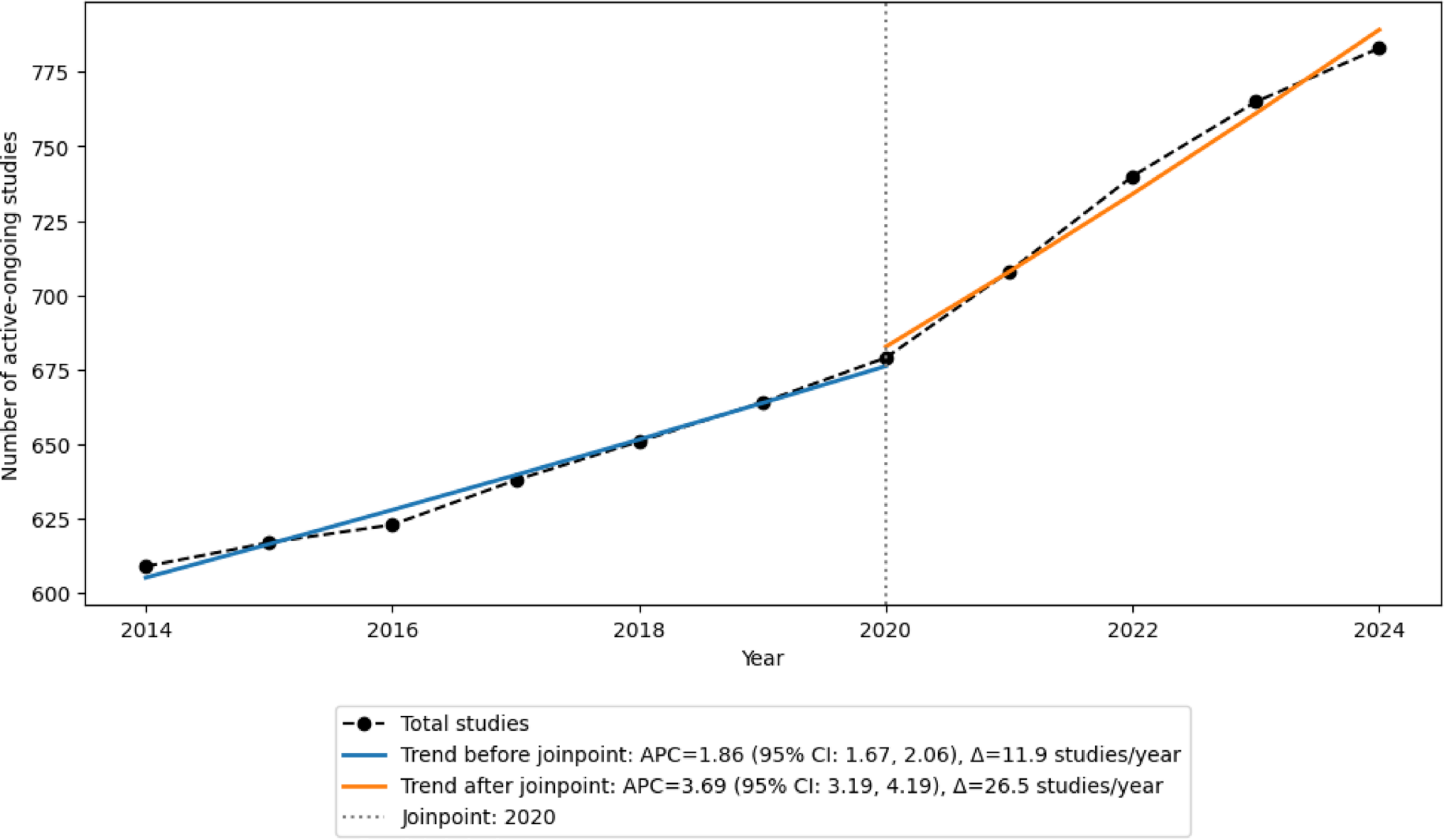
Annual trend in active ongoing clinical trials for MM (CTG, 2014–2024). *Notes*: Temporal trends in the number of active ongoing clinical trials, analyzed using joinpoint regression. ‘APC’: Annual Percent Change. APC values are shown for each segment: blue = trend before the joinpoint, orange = trend after the joinpoint. The x-axis shows the analysed year, and the y-axis represents the number of active trials. The joinpoint (vertical dotted line) indicates the year when the rate of trial initiation significantly changed

The joinpoint regression of the active ongoing trials revealed a marked acceleration in the number of trials in the last decade (Fig. 2). Before the joinpoint, at the first segment, the number of active ongoing trials increased by 1.86% annually (95% CI, 1.67–2.06), corresponding to an average increase of 11.9 studies per year. After the joinpoint, growth accelerated to 3.69% annually (95% CI, 3.19–4.19), equivalent to an increase of 26.5 ongoing trials per year. Over the entire study period, the weighted AAPC was 2.63%.

Results from negative binomial models also using CTG data showed that, compared with Phase I trials, Phase I/II trials (incidence rate ratio [IRR], 0.46; 95% CI, 0.39–0.53; *p* < .001) and Phase III trials (IRR, 0.28; 95% CI, 0.23–0.34; *p* < .001) were less frequent, whereas Phase II trials did not differ significantly (IRR, 1.09; 95% CI, 0.96–1.23; *p* = .18). Calendar year was not significantly associated with the number of newly initiated trials.

For active trials, Phase II studies were more frequent than Phase I trials (IRR, 1.39; 95% CI, 1.31–1.46; *p* < .001), whereas Phase III trials remained less frequent. The number of active trials increased over time, with calendar year positively associated with trial counts and significant growth observed from 2021 onward (2024 vs. 2014: IRR, 1.29; 95% CI, 1.16–1.43; *p* < .001) (Table 3).

**Table 3:** Incidence Rate Ratios of MM trials by phase and year (CTG; 2014–2024)

Variable	Active trials		New trials		
	IRR (95% CI)	*p*-value	IRR (95% CI)	*p*-value	
*Study phase (Ref.: Phase I)*					
Phase I/II	0.6 (0.56–0.64)	**< 0.001**	0.46 (0.39–0.53)	**< 0.001**	
Phase II	1.39 (1.31–1.46)	**< 0.001**	1.09 (0.96–1.23)	0.177	
Phase III	0.42 (0.39–0.46)	**< 0.001**	0.28 (0.23–0.34)	**< 0.001**	
*Year (Ref.: 2014)*					
2015	1.01 (0.91–1.13)	0.819	0.94 (0.73–1.22)	0.645	
2016	1.02 (0.91–1.14)	0.69	0.94 (0.73–1.22)	0.645	
2017	1.05 (0.94–1.17)	0.412	1.09 (0.85–1.4)	0.486	
2018	1.07 (0.96–1.19)	0.237	1.13 (0.89–1.45)	0.316	
2019	1.09 (0.98–1.22)	0.123	1.02 (0.79–1.31)	0.897	
2020	1.11 (1–1.24)	0.051	1.16 (0.91–1.48)	0.236	
2021	1.16 (1.04–1.3)	**0.006**	1.18 (0.92–1.5)	0.192	
2022	1.22 (1.09–1.35)	**< 0.001**	1.12 (0.87–1.43)	0.378	
2023	1.26 (1.13–1.4)	**< 0.001**	1.03 (0.8–1.33)	0.797	
2024	1.29 (1.16–1.43)	**< 0.001**	0.91 (0.7–1.18)	0.466	

Negative Binomial Regression was used to model the number of active multiple myeloma trials. The reference year was 2014, and the reference trial phase was Phase I. IRR, Incidence rate ratio; 95% CI, 95% Confidence Interval; MM; Multiple Myeloma

*p*-value < 0.05 indicates statistical significance of the association between trial phase or year and trial count

### Emerging Therapies in MM: A Horizon Scanning Approach Based on Clinical Trials

Following dataset merging and deduplication, 360 unique active ongoing clinical trials were identified for inclusion in the final analysis. Characterizing the overlap between the registries, 224 trials were present in both databases, while 131 trials were uniquely identified in CTG. These trials were associated with 271 drug and biologic candidates in active development. Most studies were concentrated in early phases, with Phase I trials accounting for 182 entries and showing substantial overlap across the databases, followed by 96 Phase I/II trials. A smaller proportion of studies were Phase III trials (*n* = 9), all of which were captured by both registries and met the inclusion criteria for detailed evaluation. Additional insights concerning the nature of the agents and routes of administration are delineated in Table 4.

**Table 4:** Overview of unique clinical trials involving MM novel therapies (2014–2024)

Clinical trial characteristics	Number (%)
	Unique trials (*N* = 360)
*Study phase*	
Early phase	32 (8.7)
Phase I	182 (49.9)
Phase I/ II	96 (26.3)
Phase II	41 (11.2)
Phase III	9 (2.5)
*Nature*	
Biologic drug	262 (71.8)
Small drug	103 (28.2)
*Route of administration*	
Oral	77 (21.1)
Parenteral	288 (78.9)

Unique trials refer to studies identified following dataset merging of ClinicalTrials.gov (CTG) and Cortellis Drug Discovery Intelligence (CDDI) and deduplication based on National Clinical Trial (NCT) identifiers; only active ongoing studies involving new investigational agents were retained for this analysis

**Fig. 3:**
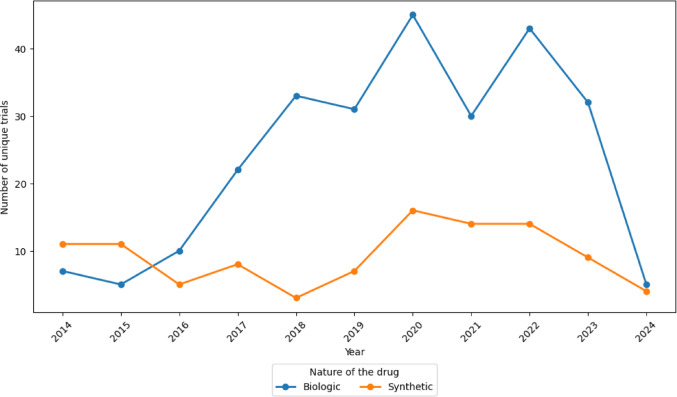
Annual Distribution of Clinical Trials by Drug Nature (Biologic vs. Synthetic) for Multiple Myeloma (2014–2024). *Notes*: Biologic therapies include cell-based interventions, such as CAR-T and TCR-engineered T cells, and T-cell–engaging antibodies. Synthetic therapies include cereblon E3 ligase modulators (IMiDs/CELMoDs) and other targeted small-molecule agents. The x-axis shows the year of study initiation, while the y-axis indicates the number of trials initiated in that year

Cell-based therapy, based on genetically modified or expanded immune cells to directly target myeloma cells emerged as the predominant intervention modality, representing 46.8% of trials (*n* = 171), followed by targeted small-molecule therapies, such as proteasome inhibitors, exportin-1 inhibitors and HDAC inhibitors, with 31.2% of trials (*n* = 114) and T-cell–engaging antibodies with 16.1% (*n* = 59). The annual distribution of the therapies by nature (biologic vs. synthetic drugs) is illustrated in Fig. 3.

**Fig. 4:**
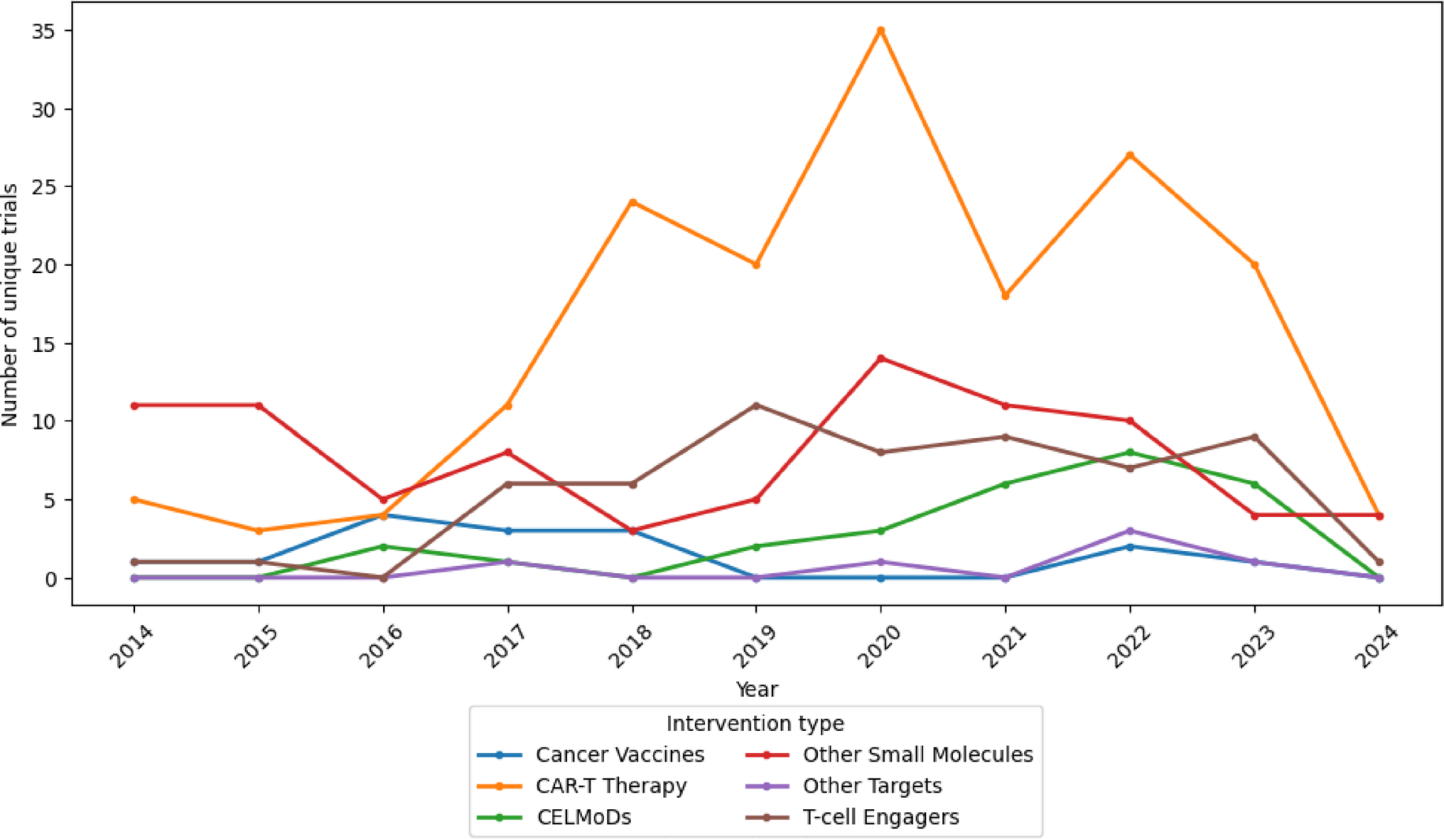
Annual Number of Clinical Trials by Therapeutic Category for Multiple Myeloma (2014–2024). Notes: ‘Cancer vaccines’: immunotherapies stimulating anti-myeloma responses, mainly dendritic cell–based; ‘CAR-T’: Chimeric Antigen Receptor T cells; ‘CELMoDs’: Cereblon E3 ligase modulators; ‘Other Small Molecules’: synthetic drugs targeting intracellular pathways (e.g., proteasome inhibitors, exportin-1 inhibitors, histone deacetylase inhibitors); ‘Other Targets’: Therapies directed against molecular targets not included in the main categories, such as specific proteins (e.g., antisense oligonucleotide targeting the interferon regulatory factor 4 mRNA); ‘T-cell Engagers’: antibodies redirecting or activating T cells (e.g., bispecific antibodies). The x-axis shows the year of study initiation, while the y-axis indicates the number of trials initiated in that year

**Fig. 5:**
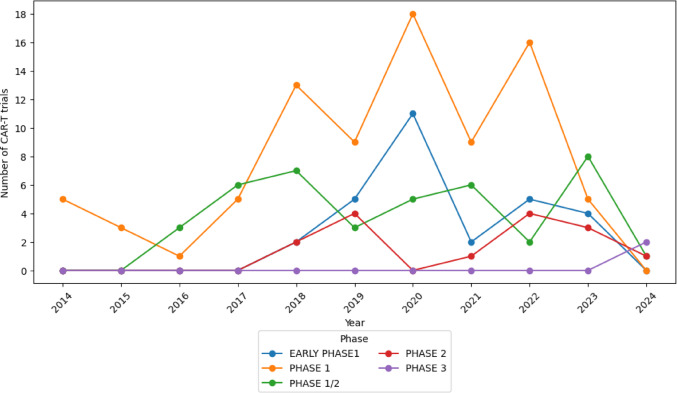
Distribution of active CAR-T Trials for Multiple Myeloma by Phase and Year (2014–2024). *Notes*: ‘CAR-T’: Chimeric Antigen Receptor T cells. The x-axis shows the year of study initiation, while the y-axis indicates the number of trials initiated in that year

Fig. 4 illustrates the annual number of interventions registered for each therapeutic category, highlighting the relative distribution across modalities. Chimeric antigen receptor T-cell (CAR-T) therapies targeting B-cell maturation antigen (BCMA; tumor necrosis factor receptor superfamily member 17, TNFRSF17, also known as CD269) accounted for a substantial proportion of cell-based approaches, representing the most frequently targeted epitope (*n* = 107) . Other commonly investigated targets included B-lymphocyte antigen CD19 (*n* = 16), and G-protein coupled receptor family C group 5 member D (GPRC5D; *n* = 12). Additional antigens of therapeutic interest encompassed SLAM family member 7 (SLAMF7; CS1), syndecan-1 (SDC1; SYND1; CD138), and CD38 (ADP-ribosyl cyclase/cyclic ADP-ribose hydrolase 1). To further illustrate the trajectory of these specific interventions, Fig. 5 presents the temporal evolution of active CAR-T trials, stratified by phase. Early-phase studies, particularly Phase I and Phase I/II, predominated throughout the study period, with notable peaks around 2018–2020 and again in 2022, reflecting sustained exploratory activity.

Among the identified antibody-based therapies, 28 trials investigated monospecific antibodies, 20 trials focused on bispecific monoclonal antibodies (BsMAbs), 9 trials explored antibody-drug conjugates, and only 3 trials examined new trispecific constructs. Although vaccines accounted for a smaller subset of trials (*n* = 15), peptide-based and DCs-based platforms predominated among the vaccine candidates. Other targeted therapies involved protein-, or oligonucleotide-based therapies with 6 trials identified.

Among the clinical trials involving small drugs as potential therapeutic agents for MM, the most frequent class was signal transduction modulators (*n* = 39), targeting pathways such as phosphatidylinositol 3-kinase/protein kinase B (PI3K/AKT), Janus kinase/signal transducer and activator of transcription (JAK/STAT), mitogen-activated protein kinase (MAPK), and apoptotic regulators including B-cell lymphoma 2 (BCL-2) family proteins and myeloid cell leukemia-1 (Mcl-1), followed by cereblon (CRBN) modulators (*n* = 28). As shown in Table 5, a substantial proportion of these agents are currently being evaluated in Phase II trials. Other less common targets in investigation were epigenetic modifiers, histone deacetylase (HDAC) inhibitors with selective inhibition of HDAC1-4, HDAC6, and HDAC10, proteasome inhibitors, and exportin-1 antagonists and alkylating agents.

**Table 5:** Ongoing phase II clinical trials of emerging therapies for RRMM identified in CTG and CDDI databases

Therapy name	NCT- identifier	Trial submission date	Sponsor	Mechanism of action	Target	Primary endpoint
BCD-248	NCT06668792	2024-10-30	Biocad	Bispecific T-cell Engager	B-cell maturation antigen (BCMA) and activate the T-cell surface glycoprotein CD3 complex	ORR
Iberdomide; (CC-220)	NCT06518551	2024-07-18	Bristol-Myers Squibb; Celgene	Protein Cereblon (CRBN) Modulators	Zinc Finger Protein Aiolos (IKZF3) Degradation Inducers; DNA-Binding Protein Ikaros (IKZF1)	DLT; MTD; PFS
	NCT05896228	2023-05-31	University of Miami	Protein Cereblon (CRBN) Modulators	Zinc Finger Protein Aiolos (IKZF3) Degradation Inducers; DNA-Binding Protein Ikaros (IKZF1)	MRD
	NCT04392037	2020-05-13	Amsterdam UMC	Protein Cereblon (CRBN) Modulators	Zinc Finger Protein Aiolos (IKZF3) Degradation Inducers; DNA-Binding Protein Ikaros (IKZF1)	PFS
	NCT04998786	2021-08-04	Nantes University Hospital	Protein Cereblon (CRBN) Modulators	Zinc Finger Protein Aiolos (IKZF3) Degradation Inducers; DNA-Binding Protein Ikaros (IKZF1)	VGPR
	NCT05177536	2021-12-15	University of Nebraska	Protein Cereblon (CRBN) Modulators	Zinc Finger Protein Aiolos (IKZF3) Degradation Inducers; DNA-Binding Protein Ikaros (IKZF1)	Treatment completion (≥ 1 year)
	NCT05354557	2022-04-26	Memorial Sloan Kettering Cancer Center	Protein Cereblon (CRBN) Modulators	Zinc Finger Protein Aiolos (IKZF3) Degradation Inducers; DNA-Binding Protein Ikaros (IKZF1)	CR
	NCT05527340	2022-08-29	PETHEMA Foundation	Protein Cereblon (CRBN) Modulators	Zinc Finger Protein Aiolos (IKZF3) Degradation Inducers; DNA-Binding Protein Ikaros (IKZF1)	ORR
	NCT05272826	2022-02-14	anadian Myeloma Research Group	Protein Cereblon (CRBN) Modulators	Zinc Finger Protein Aiolos (IKZF3) Degradation Inducers; DNA-Binding Protein Ikaros (IKZF1)	sCR
PHE885	NCT05172596	2021-12-22	Novartis Pharmaceuticals	Chimeric Antigen Receptor-Modified T Cells (CAR T Cells)	B-cell maturation antigen (BCMA)	ORR
Zevor-cel (CT053)	NCT03915184	2019-04-03	CARsgen Therapeutics Co., Ltd.	Chimeric Antigen Receptor-Modified T Cells (CAR T Cells)	B-cell maturation antigen (BCMA)	Adverse events; MTD; ORR
Mezigdomide	NCT06627751	2024-10-02	Roswell Park Cancer Institute	Protein Cereblon (CRBN) Modulators	Zinc Finger Protein Aiolos (IKZF3) Degradation Inducers; DNA-Binding Protein Ikaros (IKZF1)	ORR
Anitocabtagene-autoleucel	NCT05396885	2022-05-12	Gilead Sciences	Chimeric Antigen Receptor-Modified T Cells (CAR T Cells)	B-cell maturation antigen (BCMA)	ORR
CAR-T treatment	NCT04162119	2019-11-11	Chinese PLA General Hospital	Chimeric Antigen Receptor-Modified T Cells (CAR T Cells)	B-cell maturation antigen (BCMA) and Programmed Cell Death 1 Protein (PD-1)	Adverse Events
	NCT03931421	2019-04-25	Hospital of Zhejiang University	Chimeric Antigen Receptor-Modified T Cells (CAR T Cells)	B-cell maturation antigen (BCMA)	Adverse events; CR
	NCT05594797	2022-10-18	Hrain Biotechnology Co., Ltd.	Chimeric Antigen Receptor-Modified T Cells (CAR T Cells)	B-cell maturation antigen (BCMA)	ORR
	NCT05846737	2023-04-27	Institute of Hematology & Blood Diseases Hospital, China	Chimeric Antigen Receptor-Modified T Cells (CAR T Cells)	B-cell maturation antigen (BCMA)	MRD; safety and tolerability
	NCT05509530	2022-08-02	Xuzhou Medical University	Chimeric Antigen Receptor-Modified T Cells (CAR T Cells)	B-cell maturation antigen (BCMA) and G-Protein Coupled Receptor Family C Group 5 Member D (GPRC5D)	Adverse events
	NCT06196255	2023-12-25	Xuzhou Medical University	Chimeric Antigen Receptor-Modified T Cells (CAR T Cells)	Fc Receptor-Like Protein 5 (FCRL5)	Adverse events
	NCT05712083	2023-01-31	Zhejiang University	Chimeric Antigen Receptor-Modified T Cells (CAR T Cells)	B-cell maturation antigen (BCMA)	Adverse events; DLT
Cevostamab	NCT05801939	2023-03-24	University of Pennsylvania	Bispecific T-cell Engager	T-cell Surface Glycoprotein CD3 (CD3) and Fc Receptor-Like Protein 5 (FCRL5)	MDR; CR
MLN0128	NCT06385496	2017-03-12	National Cancer Institute (NCI)	Inhibitor of the mechanistic target of rapamycin (mTOR)	Mechanistic target of rapamycin complex 1 and 2 (mTORC1/2)	ORR
Chidamide	NCT03605056	2018-07-21	Shanghai Zhongshan Hospital	Histone deacetylase inhibitor	Histone Deacetylases	ORR
Ipatasertib	NCT06400251	2024-05-03	National Cancer Institute (NCI)	Inhibitor of protein kinase B	Protein kinase B alpha, beta and gamma (AKT1/2/3)	ORR
GSK2857916	NCT04802356	2021-03-12	PETHEMA Foundation	Antibody-Drug Conjugate	B-cell maturation antigen (BCMA)	Adverse events
	NCT04126200	2019-10-11	GlaxoSmithKline	Antibody-Drug Conjugate	B-cell maturation antigen (BCMA)	Adverse events; ORR
	NCT05847569	2023-04-27	Mayo Clinic	Antibody-Drug Conjugate	B-cell maturation antigen (BCMA)	Adverse events
	NCT05789303	2023-03-23	University of Chicago	Antibody-Drug Conjugate	B-cell maturation antigen (BCMA)	VGPR; ORR
	NCT05922501	2023-06-19	Massachusetts General Hospital	Antibody-Drug Conjugate	B-cell maturation antigen (BCMA)	ORR
GR1803	NCT06566547	2024-08-20	Hospital of Zhejiang University	Bispecific T-cell Engager	B-cell maturation antigen (BCMA) and activate the T-cell surface glycoprotein CD3 complex	ORR
Arlocabtagene Autoleucel	NCT06297226	2024-02-28	Juno Therapeutics, Inc	Chimeric Antigen Receptor-Modified T Cells (CAR T Cells)	G-Protein Coupled Receptor Family C Group 5 Member D (GPRC5D)	BOR; PR
JNJ-68,284,528	NCT04133636	2019-10-18	Janssen Research & Development, LLC	Chimeric Antigen Receptor-Modified T Cells (CAR T Cells)	B-cell maturation antigen (BCMA)	MDR
bb2121	NCT03601078	2018-07-17	Celgene	Chimeric Antigen Receptor-Modified T Cells (CAR T Cells)	B-cell maturation antigen (BCMA)	ORR; CR
C-CAR088	NCT05521802	2022-08-28	Shanghai AbelZeta Ltd.	Chimeric Antigen Receptor-Modified T Cells (CAR T Cells)	B-cell maturation antigen (BCMA)	Adverse events; ORR
Mezigdomide	NCT06627751	2024-10-02	Roswell Park Cancer Institute	Protein Cereblon (CRBN) Modulators	Zinc Finger Protein Aiolos (IKZF3) Degradation Inducers; DNA-Binding Protein Ikaros (IKZF1)	ORR; Clinical benefit rate
Ulixertinib	NCT06400225	2024-05-03	National Cancer Institute (NCI)	Inhibitor of extracellular signal-regulated kinases (ERK1/2)	Extracellular signal-regulated kinase 1 (ERK1/MAPK3) and ERK2 (MAPK1)	ORR
MLN0128	NCT06390865	2017-03-12	National Cancer Institute (NCI)	Inhibitor of the mechanistic target of rapamycin (mTOR)	Mechanistic target of rapamycin complex 1 and 2 (mTORC1/2)	ORR
Defactinib	NCT04439331	2020-06-18	National Cancer Institute (NCI)	Inhibitor of focal adhesion kinase	Protein tyrosine kinase 2 (focal adhesion kinase)	ORR
AZD1775	NCT04439227	2020-06-18	National Cancer Institute (NCI)	Inhibitor of WEE1 kinase	WEE1 G2 checkpoint kinase	ORR
GSK2636771	NCT04439188	2020-06-18	National Cancer Institute (NCI)	Inhibitor of phosphoinositide 3-kinase beta	Phosphatidylinositol-4,5-bisphosphate 3-kinase catalytic subunit beta (PIK3CB)	ORR
Taselisib	NCT04439175	2020-06-18	National Cancer Institute (NCI)	inhibitor of phosphoinositide 3-kinase	PI3K catalytic subunit alpha (PIK3CA), delta (PIK3CD), gamma (PIK3CG)	ORR

BOR, Best overall response; CR, Complete response; DLT, Dose-limiting toxicity; ERK2 (MAPK1), Extracellular Signal-Regulated Kinase 2; MRD, Minimal residual disease; MTD, Maximum tolerated dose; NCT, National clinical trial identifiers; ORR, Objective response rate; PR, Partial response; RRMM, Relapsed or refractory multiple myeloma; sCR, Stringent complete response; VGPR, Very good partial response

Figure 4 illustrates the annual number of interventions registered for each therapeutic category, highlighting the relative distribution across modalities.

Eight investigational therapies in active ongoing phase III trials for RRMM were identified across the databases, underscoring a marked discrepancy between the overall volume of late-phase MM trials and those addressing relapsed or refractory populations. The therapies include cereblon modulators (mezigdomide, iberdomide), bispecific T-cell engagers (etentamig, linvoseltamab, SG-301, felzartamab), and CAR-T cell therapies (anitocabtagene autoleucel, arlocabtagene autoleucel), targeting key molecules such as BCMA, GPRC5D, CD38, IKZF1, and IKZF3. The trial details are summarized in Table 6.

**Table 6:** Ongoing phase III clinical trials of emerging therapies for RRMM identified in CTG and CDDI databases

Therapy name	NCT- identifier	Trial submission date	Sponsor	Mechanism of action	Target	Primary endpoint
Mezigdomide	NCT05552976 NCT05519085	2022-09-21 2022-08-25	Bristol-Myers Squibb; Celgene	Protein Cereblon (CRBN) Modulators	Zinc Finger Protein Aiolos (IKZF3) Degradation Inducers; DNA-Binding Protein Ikaros (IKZF1)	PFS
Etentamig	NCT06158841	2023-11-28	AbbVie	Bispecific T-cell engager	B-cell maturation antigen (BCMA) and activate the T-cell surface glycoprotein CD3 complex.	PFS; ORR
Linvoseltamab	NCT05730036	2023-02-06	Regeneron Pharmaceuticals	Bispecific T-cell engager	B-cell maturation antigen (BCMA) and activate the T-cell surface glycoprotein CD3 complex.	PFS
Anitocabtagene autoleucel	NCT06413498	2024-05-09	Arcellx ; Kite Pharma	Chimeric Antigen Receptor-Modified T Cells (CAR T Cells)	B-cell maturation antigen (BCMA)	PFS
Arlocabtagene autoleucel	NCT06615479	2024-09-24	Juno Therapeutics, Inc.	Chimeric Antigen Receptor-Modified T Cells (CAR T Cells)	G-Protein Coupled Receptor Family C Group 5 Member D (GPRC5D)	PFS; MRD; CR
Iberdomide	NCT04975997	2021-07-02	Bristol-Myers Squibb; Celgene	Protein Cereblon (CRBN) Modulators	Zinc Finger Protein Aiolos (IKZF3) Degradation Inducers; DNA-Binding Protein Ikaros (IKZF1)	PFS; MRD; CR
SG-301	NCT06508983	2024-07-05	Hangzhou Sumgen Biotech Co., Ltd.	T-cell engager	Anti-CD38 (ADP-Ribosyl Cyclase/Cyclic ADP-Ribose Hydrolase 1)	Adverse events; Recommended stage 2 dose; PFS
Felzartamab	NCT03952091	2019-05-14	TJ Biopharma Co., Ltd.	T-cell engager	Anti-CD38 (ADP-Ribosyl Cyclase/Cyclic ADP-Ribose Hydrolase 1)	PFS

CR, Complete response; MRD, Minimal residual disease; NCT, National clinical trial identifiers; ORR, Objective response rate; PFS, Progression free survival; RRMM, Relapsed or refractory multiple myeloma

## Discussion

The present study demonstrated a substantial evolution in the scope and complexity of clinical research in MM over the past decade. Through a comprehensive and methodologically rigorous evaluation, we identified investigational agents currently in active development for MM treatment. Between 2014 and 2024, active ongoing clinical trials showed a significant upward trend (AAPC = 2.63%), with joinpoint analysis revealing an acceleration from 1.86% annual growth before the inflection point to 3.69% per year thereafter, reflecting increasing global investment in therapeutic development for MM [8, 17].

Our analysis did not explicitly model COVID-19–specific disruptions, however, the identified joinpoint in 2020, temporally coincides with the onset of the COVID-19 pandemic, a period marked by substantial disruptions and subsequent adaptations in clinical research worldwide. While the early phase of the pandemic led to trial interruptions and delays, it also accelerated the adoption of decentralized trial designs, remote monitoring, and regulatory flexibilities [18, 19]. These structural changes may have contributed to the observed post-2020 acceleration in trial growth, although a direct causal relationship cannot be established and the results should be interpreted cautiously.

MM is characterized by an immunosuppressive tumor microenvironment, thereby promoting immune evasion, drug resistance and disease progression, making MM a compelling target for ongoing and future research. Our findings revealed that cell-based therapies, particularly CAR-T cells targeting BCMA and GPRC5D, have emerged as leading investigational approaches. These results, align with recent reports underscoring the transformative impact of targeted cellular immunotherapies in MM treatment [10, 20].

The therapeutic landscape of MM has diversified considerably. Among the phase III trials identified, four distinct therapeutic classes emerged, each representing key advances in the treatment of RRMM. Cell-based approaches, particularly CAR-T therapies targeting BCMA, gained prominence due to their specificity and ability to circumvent immune escape mechanisms, yielding unprecedented response rates in hematologic cancers [8, 10, 21].

Emerging CAR T-cell products, such as anitocabtagene autoleucel and arlocabtagene autoleucel, have attracted growing interest [10, 20]. Anitocabtagene targets BCMA using a synthetic D-domain antigen-binding region engineered to reduce immunogenicity and enhance surface stability. Conversely, arlocabtagene targets GPRC5D, a novel antigen selectively expressed on malignant plasma cells. Notably, the ongoing phase III iMMagine-3 trial, identified in this analysis, is enrolling patients with RRMM who have received one to three prior lines of therapy, aiming to compare anitocabtagene with pomalidomide-, daratumumab-, or carfilzomib-based regimens [22]. The outcomes of this and other Phase III trials are highly anticipated. They are expected to further inform the evolving treatment paradigm [10].

Complementing the innovative therapeutic strategies highlighted in our horizon scan of phase III trials, bispecific T-cell engagers such as etentamig and linvoseltamab have emerged as promising agents by simultaneously targeting BCMA on myeloma cells and CD3 on T cells. This dual targeting promotes immune synapse formation and enables cytotoxic T lymphocytes to eliminate tumor cells independently of major histocompatibility complex presentation [23]. Linvoseltamab, for instance, achieved a median response duration of 29.4 months at a 200 mg dose in patients with RRMM, highlighting its potential for heavily pretreated populations [24]. Others bispecific T-cell engagers targeting CD38, such as SG-301 and felzartamab, offer novel strategies that harness adaptive immunity without relying on innate immune effector functions, addressing therapeutic resistance and expanding treatment options.

Novel cereblon E3 ligase modulators (CELMoDs), including mezigdomide (CC-92480) and iberdomide (CC-220), have gained attention in both newly diagnosed and RRMM settings. These agents enhance antitumor activity by promoting degradation of transcription factors essential for myeloma cell survival [23]. Specifically, these compounds induce the ubiquitination and degradation of IKZF1 and IKZF3, resulting in impaired plasma cell proliferation and reduced pro-survival cytokine signaling. Mezigdomide has also been shown to increase interleukin-2 and interferon-gamma production, enhance T-cell activation, and exert synergistic cytotoxicity when combined with agents like dexamethasone or bortezomib [25–27].Similarly, iberdomide exhibits a binding affinity to cereblon approximately 20-fold greater than lenalidomide or pomalidomide, with favorable oral bioavailability and pharmacokinetics that support its integration into combination regimens [28–30].

A growing number of early-phase studies are also expanding the repertoire of cell types employed, including the incorporation of NK cells and DCs [31, 32]. Among these, DC-based cancer vaccines stand out as a safe and promising approach to enhance tumor-specific immunity. However, clinical responses have been inconsistent, prompting efforts to optimize efficacy by combining DC-based therapies with conventional treatments such as chemotherapy, immunomodulatory drugs, and immune checkpoint inhibitors [31, 33, 34].

Despite significant innovation, the modest number of advanced-phase trials in the context of relapsed and refractory disease identified in our study may highlight challenges in translating early-phase successes into standard-of-care treatments. Many innovative therapies encounter obstacles during this transition due to complex manufacturing, toxicity, and logistical barriers. These challenges are particularly pronounced for cellular and immune-based therapies, limiting scalability and real-world implementation, particularly in resource-limited settings where affordable and easily distributed therapies are essential, further restricting the adoption of advanced treatments into clinical practice [35].

Consistent with prior literature, government and academic institutions played an important role as sponsors, especially in early-phase studies and in research on rare or less commercially attractive conditions, such as MM [23]. It should be noted that registry-defined sponsorship reflects regulatory responsibility rather than funding; therefore, industry involvement in investigator-initiated trials, through provision of investigational products or financial support, may be underrepresented in our analyses, with cross-national regulatory differences further contributing to variability across trial phases. Nonetheless, industry is generally more involved in later-phase trials focused on efficacy confirmation and regulatory approval, which tend to be fewer in number, a pattern that may also be reflected in our results.

The predominance of open-label designs, phase I and II trials, along with the growing proportion of combined-phase studies, and single-group assignment models reflect common trends in cancer research. The increased prevalence of single-arm studies may partly result from the adoption of accelerated approval pathways by regulatory agencies, such as the FDA since 1992 [36]. This regulatory mechanism allows the use of surrogate endpoints or smaller patient cohorts for serious or life-threatening conditions, especially in rare diseases like MM, where alternative treatments are limited [37]. Consequently, it has facilitated faster access to innovative therapies while shaping the current research landscape.

Additionally, the observed increase in the number of active studies, alongside a decline in newly initiated trials, may reflect several interrelated factors. One plausible explanation is that a substantial proportion of the active studies are later-stage trials, such as Phase II or III studies, which typically require longer enrollment periods and extended follow-up. As a result, these studies remain classified as “active” for prolonged durations, even as fewer new trials are launched. The reduction in newly initiated studies may also be influenced by shifting research priorities, limitations in resources, or operational delays that can slow study initiation. Furthermore, external factors, such as fluctuations in funding availability, or the emergence of competing therapeutic options, may impact the rate at which new studies are started [38].

Overall, the increasing focus on innovative therapeutic strategies for MM, particularly the growing prominence of biologics such as immunotherapies and gene therapies like CAR-T cells, may be related to the maturation of these technologies while also highlighting the need for more effective and personalized treatment options. In this context, continued investment in research and improved global coordination in trial registration and reporting will be critical to fully realize the potential of these emerging therapies.

## Limitations

This study presented several noteworthy limitations that must be considered when interpreting the findings. One of the primary challenges relates to potential biases in data sources, particularly due to the underrepresentation of clinical trials conducted in certain regions or countries. While the use of both CDDI and CTG broadened the global scope of the analysis, integration of data from other major registries, such as the European Union Drug Regulating Authorities Clinical Trials Database (EudraCT), Japanese Primary Registries Network (JPRN), and Chinese Clinical Trial Registry (ChiCTR), remained limited due to the lack of automated extraction tools, as their content was only available in plain text format. Similarly, although Brazil, in Latin America, provides public access to information on clinical trials through Brazilian Health Regulatory Agency’s official website, the absence of a structured database interface with export functionalities also limited its integration into the present analysis. This constraint hindered the inclusion of these datasets in our analyses, potentially narrowing the global scope of our findings. Additionally, we were not able to assess factors influencing the geographic and temporal distribution of MM trials.

Another important challenge was the reliance on proprietary curation in the CDDI database, which introduces further uncertainty, especially when source data are incomplete or inconsistently reported [39]. Similarly, variability and lack of standardization in registry reporting may have affected the completeness and accuracy of the retrieved trial data. These inconsistencies also limited our ability to consistently identify unique investigational medical products across trials. Although manual verification by two independent reviewers improved data quality, it could not fully address structural gaps in reporting. To mitigate this limitation, CTG records were systematically cross-referenced with the CDDI database using the National Clinical Trial identifiers to recover missing information whenever possible. However, residual information bias may affect the results. The higher number of CAR-T studies observed should also be interpreted with caution. In some regions, the decentralized manufacturing model of these therapies may lead to individual sites being registered as separate studies, which could inflate trial counts compared to other therapeutic modalities. As this aspect was not specifically assessed in our analysis, its potential impact on our findings remains uncertain. Additionally, our focus on innovative compounds excludes supportive care, reformulations, and label-extension studies, which may limit the generalizability of our findings to the broader MM clinical trial landscape and underestimate the number of trials. Finally, this analysis did not explicitly model COVID-19–specific disruptions, as a result, residual pandemic-related effects may not be fully captured by the available data. Although the joinpoint identified in 2020 coincides with the onset of the COVID-19 pandemic, this temporal overlap should be interpreted cautiously. The observed change in trend likely reflects a combination of factors, including long-term advances in therapeutic development and evolving clinical trial infrastructures, rather than the effect of a single external event.

## Conclusion

In conclusion, our study provides valuable insights into the evolving therapeutic landscape of MM by highlighting current trends in clinical development and the growing focus on innovative modalities such as immunotherapies and gene therapies. By mapping the distribution and characteristics of active phase III trials, the study contributes to the efforts in horizon scanning and strategic planning for future healthcare decisions. Furthermore, it underscores the importance of enhancing data transparency and standardization across clinical trial registries to better support evidence-based policy and equitable access to emerging treatments.

In the near term, pivotal phase III trials of CAR-T cells, bispecific antibodies, and CELMoDs are expected to shape updates to standard-of-care regimens for RRMM, expanding therapeutic options across multiple lines of treatment. Ongoing research should prioritize strategies to optimize efficacy, mitigate toxicities, and ensure equitable global access to these emerging therapies.

## Data Availability

The datasets supporting the findings of this study are available from the corresponding author, upon reasonable request.
